# Stroke Survivor and Caregiver Perspectives on Post-Stroke Visual Concerns and Long-Term Consequences

**DOI:** 10.1155/2018/1463429

**Published:** 2018-10-04

**Authors:** Theresa M. Smith, Monique R. Pappadis, Shilpa Krishnan, Timothy A. Reistetter

**Affiliations:** ^1^Department of Occupational Therapy, Texas Woman's University, 6700 Fannin Street, Houston, TX 77030, USA; ^2^Division of Rehabilitation Sciences, School of Health Professions, University of Texas Medical Branch, 301 University Blvd, Galveston, TX 77555, USA; ^3^Sealy Center on Aging, University of Texas Medical Branch, 301 University Blvd, Galveston, TX 77555, USA; ^4^Department of Rehabilitation Medicine, Division of Physical Therapy, Emory University, School of Medicine, 1462 Clifton Road, Atlanta, GA 30322, USA; ^5^Department of Occupational Therapy, School of Health Professions, University of Texas Medical Branch, Galveston, 301 University Blvd, Galveston, TX 77555, USA

## Abstract

Approximately 800,000 people in the United States have a stroke annually. Up to two thirds of stroke survivors have some visual problems, which result in disability and can affect survivors' overall rehabilitation outcomes. Although some post-stroke visual impairments can be corrected and respond well to intervention, ocular signs can be subtle and may not be recognized or reported by the stroke survivor but rather by a vigilant caregiver. The purpose of this study was to explore the post-stroke visual concerns and consequences expressed by stroke survivors and caregivers. This study employed a qualitative design using semistructured interviews conducted with a convenience sample of stroke survivors and caregivers recruited from either a community support group or skilled nursing and long-term care facilities. Interviews were recorded and transcribed verbatim. Comparative content analysis was used to identify vision-related themes by two independent coders. All research team members completed quality checking of coding. Twenty participants (11 stroke survivors and 9 caregivers) expressed visual concerns or consequences following stroke: (1) eye movement problems, (2) perceptual issues, and (3) consequences of vision problems or issues, which affected their daily life/quality of life. Stroke survivors and caregivers reported receiving vision care from (1) eye doctors, (2) occupational therapists, and (3) other healthcare professionals. All vision care providers need to be observant of potential post-stroke visual concerns. Stroke survivors should have a thorough vision evaluation to optimize their independence in everyday activities and quality of life.

## 1. Introduction

Approximately 800,000 people in the United States have a stroke annually [1]. With the rising population of older adults, the incidence of stroke is expected to increase and survival rates of stroke continue to rise [2]. Up to two thirds of stroke survivors have some visual problems that result in disability [3]. A wide range of visual impairments can occur following stroke including low vision, eye movement and visual field abnormalities, and visual perceptual difficulties [4, 5]. These impairments invariably affect survivors' independence in daily living and quality of life, negatively influencing their ability to drive, work, and their confidence and familial relationships [6].

Stroke survivors reported low vision acuity for near and distance vision [4]. Nearly 20–60% of stroke survivors have permanent or resolving visual field deficits usually homonymous hemianopia or quadrantanopia, and 70% may be affected by eye movement disorders [7]. Eye movement disorders can result in a loss of depth perception, reduced eye-hand coordination, problems with scanning, and difficulties reading [7]. Strabismus, a misalignment of the eyes, can affect stroke survivors with resulting complaints of diplopia [8]. Visuospatial inattention, also termed visual neglect or hemispatial neglect, can occur following right hemisphere stroke [9]. Other post-stroke visual concerns pertain to eye-hand coordination deficits. These deficits have been shown to result in reduced independence and quality of life [10]. Other conditions that can lead to decreased visual acuity such as glaucoma, cataract, retinal diseases, macular degeneration, and refractive errors [10].

Visual impairments are not as evident as motor or speech impairment [11] and without thorough screening subtle visual complications may go undiagnosed. In the United States, visual acuity is commonly assessed with a Snellen chart [12, 13] and visual fields by the Humphrey visual field [14]. Visual field deficits may be screened using a confrontation test. Most eye movement disorders can be detected during a clinical evaluation [15]. Paper and pencil tasks are used routinely to assess visual perceptual symptoms of visual inattention even though they may not detect symptoms in a chronic stage [16].

Prior studies have identified patients with significant vision impairments, many of whom did not have proper corrective lens following a stroke. One study found that individuals admitted for rehabilitation following stroke usually do not have their glasses with them or have glasses that were not in acceptable condition [17]. Even among those with existing glasses, some still had impaired vision.

Ninety-two percent of stroke survivors with suspected visual problems have been determined to have low vision, eye movement deficit, visual field impairment, and/or visual perceptual deficits such as visual inattention [4]. It is important to treat visual problems that can adversely affect rehabilitation outcomes of stroke survivors [5, 17, 18]. Treatment for eye movement disorders could be restorative, compensative, substitutive, pharmacological, or assessment and screening interventions [19]. Visual fields deficits may improve, but maximal improvement generally occurs within a month [18]. Treatment for visual field deficits involves training in compensatory eye movements, and some patients have prisms in their glasses. Visual inattention is commonly treated by training the patient to scan to the neglected side. Increasing more complex tasks is completed to increase attention [20]. Other treatments for visual inattention include video feedback during treatment, training in visual imagery, diplopia, and vestibular, somatosensory, and optokinetic stimulation. Prisms may also be used [18]. With rehabilitation, partial to full recovery is possible for some visual disorders secondary to stroke [11, 18, 21].

Many of these visual impairments can be corrected and respond well to intervention [18]. However, some visual impairments can be subtle and may not be recognized or reported by the patient but rather by a vigilant caregiver [5]. Some visual problems resulting from stroke such as visual field deficits can be chronic [18] or resistant to treatment. The consensus in the literature is that a thorough vision evaluation in the rehabilitation setting and targeted rehabilitation are critical to maximize patient functional performance [4, 5, 11, 18, 21]. It is important for healthcare providers to understand how stroke survivors and their caregivers experience visual concerns after stroke to ensure that care is focused on areas that matter most to those affected by stroke. Hence, the purpose of this study was to identify the vision concerns of stroke survivors and the consequences of visual impairments as perceived by stroke survivors and caregivers.

## 2. Methods

### 2.1. Research Design

In the current qualitative study, interviews of stroke survivors and caregivers were only included if they discussed aspects related to vision. This cross-sectional study is a part of a larger patient-centered outcomes research study that collected data on stroke survivors and caregivers on their preferences and needs following stroke [22]. The University's Institutional Review Board approved this study.

### 2.2. Participants and Recruitment

Participants were recruited using convenience sampling techniques from local stroke support groups as well as skilled nursing and long-term care facilities in the Houston and Galveston area in Texas. The stroke survivors were included if they were 18 years and older, self-identified with a previous diagnosis of stroke, and communicated in English. The caregivers were recruited into the study if a survivor identified them as their primary caregiver. Not all caregivers agreed to participate, and in some cases, the caregivers identified by the stroke survivors participated even if the stroke survivors themselves did not participate. The eligible participants were identified and enrolled into this study by a research coordinator with a background in psychology.

### 2.3. Data Collection

After obtaining the participant's written consent to participate, semistructured, in-person interviews were conducted. Separate, independent interviews were conducted for stroke survivors and caregivers at their preferred location [23–25]. The interview guide was developed by a multidisciplinary team involving an occupational therapist (TR) with expertise in health services and outcomes research and a social scientist with over 30 years of experience in qualitative research [25, 26]. The semistructured interview guide included the following content: description of the stroke event and consequences following stroke, therapy and services received, goals in rehabilitation, and what was most important. The interview guide for caregivers included similar content, in addition to content related to their role in the decision-making process, need for information, and advice for other caregivers. All participants were probed for clarification and additional information [27]. The interview guide did not include specific vision-related questions; however, it did include general questions to identify all perceived stroke-related symptoms or concerns.

Male and female licensed occupational (TR) and physical therapists (SK) conducted the interviews. The interviewers had prior experience in conducting semistructured interviews, qualitative methods, and rehabilitating individuals with stroke. In addition, the interviewers had no current or previous connection with any participants recruited in this study. The interviewers revealed their existing affiliations and the purpose of the study to the participants. On an average, each interview lasted for 30 minutes (range: 20–60 minutes). These interviews were audio-recorded and transcribed using professional transcription services. The participants were compensated with a $25 gift card for their time and contribution to this study.

### 2.4. Data Analysis

We used a combination of thematic and content analysis approaches to code the transcripts (Bernard and Ryan [25]). We started with open coding (i.e., identifying initial codes and categories) and then continued with axial coding (i.e., identifying relationships between the initial codes), and then proceeding with selective coding (i.e., coding any data associated with our core vision-related themes). The transcripts of the stroke survivors were read and coded independently by MP and TR for themes and subthemes related to voluntarily disclosed vision concerns and quality-checked by SK and TS for relevance and consistency. The transcripts for the caregiver interviews were read and coded independently by SK and TR and quality-checked for consistency and relevance by MP and TS. Constant comparison was done within and across transcripts to enhance trustworthiness of the coding process.

For the current study, only themes related to vision were coded and discussed. Of the 65 interviews (41 stroke survivors and 24 caregivers) from the larger study, 20 participants (11 stroke survivors and 9 caregivers) expressed themes or concepts related to vision. Any disagreements in codes were resolved by discussion with the entire research team. The benefits of utilizing multiple coders with multidisciplinary backgrounds including occupational therapy, low vision rehabilitation, physical therapy, social work, expertise in qualitative methods and analyses, and expertise treating individuals with neurological impairments helped us refine our themes from multiple perspectives and limited specific biases [25, 28]. All members of the research team had their primary appointment in an academic university with a medical center during the time of this study.

The final codebook was created, and the refined themes and subthemes were entered into a qualitative analysis software, NVivo 10 [29]. We also performed text search queries in NVivo 10 with words and concepts related to vision, including but not limited to “see,” “vision,” “read,” “eye,” “glasses,” and “perception” to make sure all concepts related to vision were coded from all interviews. Text queries also identified words with the same stem or synonyms. For example, when we queried *see*, we obtained text related to *seeing*, *read*, and *saw*. In addition, NVivo helped us organize and synthetize our themes.

The final set of themes and subthemes was formed after combining relevant subthemes and was agreed upon by all members in the research team. The themes were grouped into relevant categories to align in a consistent manner with the rehabilitation process along with supportive quotations. To ensure quality control of the data [30], we used triangulation methods for clarification and to better understand vision concerns if mentioned by both the caregiver and the stroke survivor. In addition, all members of the research team have been sufficiently engaged with stroke survivors and caregivers due to their clinical experience. Lastly, we maintained an audit trail by keeping records regarding code/theme development and team decisions regarding the analysis and interpretation of the data.

## 3. Results

During the qualitative interviews of a larger study including 65 participants, which included 40 stroke survivors and 25 caregivers, 11 stroke survivors and 9 caregivers reported visual concerns. There were four female and seven male survivors ranging in age from 42–74 years of age (mean age = 62.1, SD = 9.4). Five of the survivors were Caucasian, five were African-American, and one was Hispanic. Five survivors had inpatient rehabilitation, one of which also had home health, three had outpatient therapy, one survivor's rehabilitation was unknown, one had “other therapy,” and one had no rehabilitation. The majority of stroke survivors were 1–2 years post-stroke (*n* = 8), while the others were 3–5 years post-stroke. There were eight female caregivers and one male caregiver who ranged in age from 24 to 89 years (mean age = 66.7, SD = 18.6). Eight of the caregivers were Caucasians, and one was African American. Half of the caregivers were caring for a stroke survivor who was 1–2 years post-stroke, whereas the others were 3–5 years post-stroke. A formal caregiver cared for a number of patients; therefore, it is unclear how long post-stroke they were.

The qualitative data results are presented below. The results are based on the two main themes derived from the data, which are the visual concerns and consequences mentioned by the stroke survivors and caregivers and the vision care providers to which they attributed their care.

### 3.1. Visual Concerns and Consequences

Three subthemes of visual concerns emerged from the data including eye movement problems, perceptual issues, and consequences of these problems or issues (see Table 1). Codes representing the eye movement problems subtheme included double vision, eyes cannot focus, eyes jumping around, and eyelid does not close. Codes demonstrating the perceptual issues subtheme consisted of trouble distinguishing colors, loss of depth perception and peripheral vision, left neglect, and impaired visual processing. The third subtheme of visual concerns and consequences encompassed the following codes: reading, impaired balance and falls, incapacity to drive, and inability to work.

### 3.2. Vision Care Providers

The stroke survivors and caregivers discussed which vision care providers they saw for these visual concerns and interventions they received from the different vision care providers (see Table 2). The three named vision care providers were eye doctors, occupational therapists, and other healthcare professionals. Participants related that eye doctors were seen for examination, magnifiers, glasses, and eye patch. Occupational therapists addressed visual concerns with scanning, gaming, and eye-hand coordination activities. Other healthcare professionals included unidentified professionals who provided compensation techniques and therapy.

## 4. Discussion

The purpose of this study was to explore voluntarily disclosed visual concerns from the perspectives of the stroke survivors and caregivers. Persons with stroke and caregivers expressed how the consequences of eye movement problems and perception issues led to a decrease in occupational performance of daily activities, such as eye-hand coordination, reading, maintaining balance, driving, and working. Conjugate eye deviations and neglect commonly occur together and reduce fast eye movements needed for reading, mobility, and visual exploration [11], and visual field deficits negatively affects one's ability to drive [31].

The emergence of visual concerns from survivors and caregivers within our study is substantiated by the findings of another research study that employed similar qualitative methodologies [7]. One of the top five priorities of stroke survivors and caregivers was to learn the best ways to treat visual problems after stroke [7]. Participants in our study described specific consequences of visual problems and perceptual issues, such as difficulty reading, impaired balance and falls, incapacity to drive, and inability to work. Other researchers have studied how visual problems can affect balance and contribute to fear of falling and falls [6]. Visuospatial neglect was associated with risk for falls, but the correlation of visual field deficit and falls is less clear [32]. Postural stability is also related among other factors to visual condition of stroke survivors, and decreased visual acuity increases the risk for falls [33]. Driving is a complicated occupation requiring many skills, but sufficient visual function is fundamental as it is for work.

Both stroke survivors and caregivers conveyed the importance of specialized care for visual concerns and named specific interventions used by vision care providers. Further, they expressed frustration when they were unable to access vision care providers or felt vision concerns were overlooked. Stroke survivors in a study by Rowe also communicated that visual concerns were overlooked by medical staff members as their attention was directed to other disabilities from the stroke [6]. Caregivers as well as survivors lamented that survivors were not referred for vision rehabilitation when it was needed following stroke.

*I guess I am wishing and hoping something can be done about my eyesight … I wish I would have pushed harder on my primary care doctor to look into the eye problem, but I didn't and we just kept passing that over*.

Some participants discussed their issues with misdiagnosis of their visual impairments that resulted in them not receiving timely eye care. This misinformation frustrated the patients and their caregivers. For instance, a caregiver stated:

*First we went to the Ophthalmology … … … .and we saw doctor and he said this was probably a neurological issue for the visions so he sent us to the Neuro-Ophthalmologist doctor downtown … [the] doctor was looking for horses before zebras, so he tested [him] for myasthenia gravis and thyroid malfunction cause he felt those could be an explanation for [his] vision problems, which also included some double vision, and of course, those test came back negative. [The] doctor wanted to see his CAT scan and everything from immediately after the stroke. Unbelievably, he just saw those on the last couple of weeks even though our report was back in July … I just spoke from someone from their office today and [the doctor] said he can either come and have prism lenses and/or do some exercises for the eyes. It's taken from July 25th to literally today (December 4^th^) to find that out.*

Accurate diagnosis for visual impairments must be conducted by utilizing reliable and valid outcome tools so that these individuals are referred to appropriate rehabilitation services. Healthcare providers must discuss these concerns in their multidisciplinary team meetings which can augment the diagnosis of vision-related impairments following stroke [34]. Our data suggest that stroke survivors who received inpatient rehabilitation (IRF) were more likely to be seen by occupational therapists. Prototypical quotations stating that occupational therapy was received were attributed to two caregivers and two stroke survivors. These stroke survivors received occupational therapy in an IRF.

The emerging themes from this study illuminate a number of implications for vision care providers. Stroke rehabilitation programs may not address stroke-related vision problems unless stroke survivors report difficulty and survivors thereby go untreated [35]. To date, no standardized vision screen to accurately assess all post-stroke visual impairment exists [36]. In a recent systematic review, the authors identify two vision screening tools purported to screen for vision problems post-stroke [36]. Due to varying levels of sensitivity when administered, we argue first that all stroke survivors should be screened using a standardized performance-based functional vision screen. At the Royal London Hospital, all stroke survivors are administered a functional vision screen by occupational therapists to assess convergence, smooth pursuits, saccades, visual field deficits, and visual inattention [5] before being referred to an orthoptist for a full evaluation, including acuity testing. When compared to orthoptist findings, outcomes of the occupational therapy functional vision screen of stroke survivors were 68% analogous [5]. Routine use of a standardized vision screening tool could lead to targeted referrals to other vision care specialists and for appropriate interventions [36].

Second, it is vital that persons post-stroke be evaluated for refractive error correction or prisms as needed. All stroke survivors and caregivers strongly suggest the need for more thorough assessment of eye movement problems (i.e., double vision, eyes do not focus, eyes jumping around, and eyelid does not close) as well as perceptual issues (i.e., trouble distinguishing colors, loss of depth perception, loss of peripheral vision, left neglect, and impaired visual processing). Studies have shown that a significant number of visual deficits from stroke are undetected [4, 18].

Third, it would be advantageous for stroke survivors and caregivers to understand the behaviors or signs and symptoms that may be indicative of a visual problem and/or visual concerns. Vision care providers at all levels of care should provide education on post-stroke visual concerns to stroke survivors and caregivers that may require treatment. If these visual concerns cannot be remediated, then stroke survivors and caregivers would benefit by education on coping strategies [11].

Fourth, a straightforward linkage of diagnosed visual impairment to targeted interventions is needed [37]. Vision care providers need to know how to use evidenced-based practice to assess and treat eye movement problems and perception issues at all levels on the continuum of care to prevent and/or lessen their consequences [38]. Barriers to use of evidence-based practice for visual inattention by occupational therapists have been shown to be their lack of basic knowledge in accessing and using the evidence [39]. It is the ethical responsibility of vision care providers to ensure timely referral to other providers as indicated such as ophthalmologists, neurologists, optometrists, orthoptists, physiatrists, occupational therapists, physical therapists, low vision therapists, orientation and mobility specialists, or social workers. Last, all providers need to advocate for stroke survivors and their caregivers to receive much needed services throughout the care continuum and not be limited to IRFs.

### 4.1. Limitations of the Study

There are a number of limitations in the study. The original interview guide in the larger study did not include any direct questions on vision problems. Rather, stroke survivors and caregivers were asked to describe their post-stroke experiences. Only 20 of them discussed noticeable vision changes following stroke. This could have resulted in selection bias occurring with those 20 reporting vision problems possibly being more sensitive to vision problems, and their concerns may not be representative of other stroke survivors and caregivers. Conversely, those who did not mention vision problems may have focused on other stroke sequelae, lacked awareness of vision-related changes, or did not ultimately have vision problems. Although participants were queried on post-stroke experiences, they may not have taken into consideration premorbid visual problems or other diagnoses, such as diabetes or thyroid dysfunction, adversely affecting vision. Although vision-related questions were not probed, a large number of participants discussed these consequences—justifying the importance of the analysis conducted for this study.

Further, we did not have participants' baseline visual function and there was no optometrist or ophthalmologist involved in this study or in the assessment of the stroke survivor's visual complains. However, the first author is a certified low-vision occupational therapist with extensive experience with stroke-related vision problems. Since the majority of our participants were recruited from the community, we did not have access to clinical indicators (e.g., stroke location, type, and severity), which may confound the results and conclusions of this study. Future research is needed to explore how such clinical factors influence the development of vision-related concerns following stroke. However, our sample was diverse, and the results may guide future research and programs to address the vision-related needs of persons following stroke.

## 5. Conclusion

Findings from this study indicate that visual concerns of stroke survivors and caregivers may not be addressed sufficiently, thereby adversely affecting survivors' independence in everyday activities and quality of life. All vision care providers need to be vigilant of these unmet visual concerns and complement their care accordingly, including referring to other appropriate eye care providers. A thorough post-stroke vision evaluation is imperative to assess visual acuity, eye motility, visual fields, and visual perception. Evidence-based assessments and targeted treatments to address visual concerns are needed across the continuum of care, not limited to IRFs. Future research should focus on barriers to adhering to best practice in detecting and treating post-stroke visual concerns.

## Figures and Tables

**Table 1:** Subthemes and codes for the theme: visual concerns and consequences.

Subthemes	Code frequency	Prototypical quotations
*Eye movement problems*		
Double vision	1 ss; 3 c	“I got up to go to the bathroom and the double vision was so bad I could not make it from the bathroom to the bed.” (ss24, 68, M, IRF) “She was now having real visual problems. She was seeing double and things were happening.” (c17, 74, M, acute rehab only)
Eyes do not focus	1 ss	“Well, worse problem I have is with my eyes. They do not focus together.” (ss5, 71,M, no rehab)
Eyes jumping around	1 c	“The one thing that I do regret is that I did not jump on them more about his eye and even Dr. ^∗∗∗^ and them … I mean finally after we'd been there all through intensive care obviously they know his eyes are jumping around.” (c1, 65, F, IRF)
Eyelid does not close	1 c	“…and the idea of his eye not staying shut is one big thing. And had I known more about it I would have insisted they get somebody in there to close that eye so that it would not get hurt, the cornea.” (c1, 65, F, IRF)

*Perceptual issues*		
Trouble distinguishing colors	1 ss	“I can see colors, all kinds of colors but if you have a pink and close to a pink, I can't distinguish, or a white and a yellow. Like, if it's a light yellow, I'm using that as a for instance. I can't distinguish that anymore.” (ss23, 74, F, IRF)
Loss of depth perception	1 ss; 1 c	“My depth perception is real off.” (ss7, 57, F, acute rehab) “We lay things out and his eyes still are a problem because he cannot see the depth perception.” (c1, 65, F, IRF)
Lost peripheral vision	4 ss; 2 c	“It's my eyes. I can't see out of the peripheral…” (ss42, 67, F, IRF) “He did have peripheral vision on the right side was lost, and we noticed that weeks later we were driving down 610 and there's a Derek Hotel… he said, “What is a Derek Hot?” He didn't get the last part of it, the E-L, hotel.” (c20, 76, F, outpatient rehab)
Left neglect	3 ss; 2 c	“I was determined to have left neglect as well. If you're not familiar with that, it's a pretty bizarre condition. Someone came from behind me and startled me. I kind of was spooked, so I, “Whoa,” by the person coming around.” (ss19, 55, M, acute care rehab only) “His perception is pretty off, he has left side of negligence.” (c11, 73, F, home health)
Impaired visual processing	2 ss; 2 c	“I can see things all around me. No problem that way but when it comes to them together, I can put them together but I have to really concentrate.” (ss23, 74, F, IRF) “And a problem she has today is that she can see something but it doesn't…it happened too quickly for her to digest.” (c17,74, M, acute rehab only)

*Consequences of vision problems or issues*		
Decreased eye-hand coordination	3 c	“But the hand-eye, those that have had visual issues and coordination issues” (c17,74, acute rehab only)
Difficulty reading	3 c	“He did teach himself to read, though. He worked about five hours a day on the computer and reading.” (c20, 76, F, outpatient)
Impaired balance and falls	2 ss; 2 c	“Balance and vision? Yea…that's my main complaints.” (ss7, 57, F, acute rehab only) “You are going to fall or something else is going to happen to you if we do not get you to the doctor and get your eyes examined.” (c4, 76, F, NR)
Incapacity to drive	1 ss	“He said that I had lost peripheral vision in my left eye…I do not drive my car anymore.” (ss37, 63, M, NR)
Inability to work	1 c	“For the longest time, all he wanted to do was go back to work and he began to realize that he couldn't read properly.” (c11, 73, F, home health)

ss = stroke survivor; c = caregiver; IRF = inpatient rehabilitation facility; F = female; M = male; NR = not reported.

**Table 2:** Subthemes and codes for the theme: vision care providers.

Subthemes	Code frequency	Prototypical quotations
*Eye doctor*		
Examination	2 c	“She's actually a[n] ophthalmologist. The exam that she performed was very thorough.” (c17,74, M, home health)
Magnifiers	1 c	“The VA gave ^∗∗^ one of these magnifiers for reading, he used it for probably a year, and then…didn't need it anymore.” (c11, 73, F, home health)
Glasses	3 ss; 1 c	“I also received prism glasses from a neuro ophthalmologist in California which was very helpful.” (ss19, 55, M, outpatient) “^∗∗∗^ can either come and have prism lenses or do some exercises for the eyes.” (c2, 54, F, no rehab)
Eye patch	1 c	“An ophthalmologist…had us tape the eye shut…he did say if you were staying I think I would go ahead and stitch that eye up.” (c1, 65, F, IRF)

*Occupational therapist*		
Scanning	1 ss	“Occupational therapists showed me some techniques to …to improve on my visual scanning.” (ss36, 44, M, IRF)
Gaming	1 ss	“They [occupational therapists] gave me aids, such as apps and also games to play or something that will help with that.” (ss36, 44, M, IRF)
Eye-hand coordination	2 c	“I guess the occupational therapy focused more on the hand-eye coordination kind of stuff.” (c14, 24, F, IRF)

*Others*		
Compensation	2 ss	“They had to turn my bed so I actually look to the left” (ss19, 55, M, outpatient)
Therapy	1 ss; 1 c	“A lot of therapies…for my vision.” (ss41, 69, M, IRF & home health) “We didn't have occupational therapy because…vision comes out of our physical therapy budget, according to Medicare.” (c17,74, M, home health)

ss = stroke survivors; C = caregivers; IRF = inpatient rehabilitation facility; M = male; F = female; rehab = rehabilitation.

## Data Availability

The data used to support the findings of this study are available from the corresponding author upon request.
